# DEPDC1B is a tumor promotor in development of bladder cancer through targeting SHC1

**DOI:** 10.1038/s41419-020-03190-6

**Published:** 2020-11-17

**Authors:** Chin-Hui Lai, Kexin Xu, Jianhua Zhou, Mingrui Wang, Weiyu Zhang, Xianhui Liu, Jie Xiong, Tao Wang, Qi Wang, Huanrui Wang, Tao Xu, Hao Hu

**Affiliations:** Department of Urology, Peking University People’s Hospital, Beijing, China

**Keywords:** Bladder cancer, Cell signalling

## Abstract

Bladder cancer is one of the most commonly diagnosed malignant tumors in the urinary system and causes a massive cancer-related death. DEPDC1B is a DEP domain-containing protein that has been found to be associated with a variety of human cancers. This study aimed to explore the role and mechanism of DEPDC1B in the development of bladder cancer. The analysis of clinical specimens revealed the upregulated expression of DEPDC1B in bladder cancer, which was positively related to tumor grade. In vitro and in vivo studies showed that DEPDC1B knockdown could inhibit the growth of bladder cancer cells or xenografts in mice. The suppression of bladder cancer by DEPDC1B was executed through inhibiting cell proliferation, cell migration, and promoting cell apoptosis. Moreover, a mechanistic study found that SHC1 may be an important route through which DEPDC1B regulates the development of bladder cancer. Knockdown of SHC1 in DEPDC1B-overexpressed cancer cells could abolish the promotion effects induced by DEPDC1B. In conclusion, DEPDC1B was identified as a key regulator in the development of bladder cancer, which may be used as a potential therapeutic target in the treatment of bladder cancer.

## Introduction

Bladder cancer is a heterogeneous tumor originating from the transitional epithelium of the bladder (urethral epithelium) with at least 40 histological subtypes. The latest data from GLOBOCAN showed that bladder cancer accounts for 3% of all cancers in the world and with a high incidence, especially in developed countries^1,2^. People older than 55 years are more likely subjected to bladder cancer, which is four times even common in men than in women^3^. In addition, the 5-year survival rate of patients with bladder cancer is about 70%, whereas the 5-year survival rate of those with metastasis is only 5%^1,2^. Bladder cancer causes serious damage to patients all over the world because of its high metastasis rate and recurrence rate, as well as lack of effective treatment. Traditionally, local bladder cancer can be cured by surgical resection or radiotherapy. For recurrent or aggressive bladder cancer, more systematic treatment is required to control the disease and relieve symptoms^4,5^. The promising new therapeutic options currently under research include the use of immune-checkpoint inhibitors, antigen-drug conjugates, and targeted approaches that attack long non-coding RNAs, microRNAs, PARP1, and receptor signaling pathways^6,7^. Considering the current situation of bladder cancer, the development of new treatments has become an urgent problem to be solved^8–15^. Therefore, it is necessary to further explore the molecular mechanism of bladder cancer, to identify more effective targets.

The DEP domain protein 1B (*DEPDC1B*) gene is located on chromosome 5 (5q12.1) and encodes a protein containing two conserved domains DEP and RhoGAP. On the one hand, the DEP domain enables proteins to interact with G protein-coupled receptors and negatively charged membrane phospholipids. On the other hand, the RhoGAP domain is responsible for Rho GTPase signal transduction^16,17^. DEPDC1B can interact with a variety of signal molecules, from splicing regulators to transmembrane proteins. The expression of DEPDC1B is positively regulated by p63 and there is a p63-binding site at the 27 kb from the initiation of transcription^18^. Moreover, Pitx2 can inhibit the expression of GTPase activating protein DEPDC1B at the transcriptional level^19^. Besides, DEPDC1B is required for the coordination of deadhesion events and cell cycle processes in mitosis^20^. Furthermore, DEPDC1B promotes the growth, invasion, and anchoring-independent growth of oral cancer cells through the interaction between Rac1 and ERK protein^21^. Different levels of DEPDC1B may affect the prognosis of patients with prostate cancer through the regulation of autophagy^22^. Although the role of DEPDC1B in the progression of various types of cancers have been understood, whether DEPDC1B could affect bladder cancer remains to be elucidated.

In this study, we found that DEPDC1B was upregulated in tumor tissues of bladder cancer and related with malignant grade. Knockdown (KD) of DEPDC1B could inhibit the development and progression of bladder cancer in vitro and in vivo. Moreover, through RNA sequencing, SHC1 (Src homology 2 domain containing transforming protein 1, alias ShcA) was identified as a potential downstream of DEPDC1B, downregulation of which exhibited similar effects on bladder cancer cell functions with DEPDC1B KD and could alleviate the promotion effects of DEPDC1B overexpression on bladder cancer. These results indicated that DEPDC1B may act as a tumor promotor in the development of bladder cancer through the regulation of SHC1 and may be a novel therapeutic target for improving treatment strategy of bladder cancer.

## Material and methods

### Cell culture

Bladder cancer cell lines T24 were purchased from Cell Bank of Chinese Academy of Sciences (Shanghai, China) and EJ were obtained from BeNa Technology (Hangzhou, Zhejiang, China). T24 cells were cultured in McCOY’s 5A medium (Invitrogen, Carlsbad, CA, USA) supplemented with 10% fetal bovine serum (FBS) and EJ were cultured in 90% RPMI-1640 with 10% FBS. All culture medium was changed every 3 days. All cells were maintained at 37 °C with 5% CO_2_ and 95% humidity. All cell lines were confirmed by short tandem repeat (STR) profiling.

Bladder cancer cell lines T24 and EJ cells were transfected with Lipofectamine 3000 (Invitrogen, Carlsbad, CA, USA) according to the manufacturer’s instructions. The cell transfection efficiency was assessed by quantitative reverse transcriptase PCR (qRT-PCR) after 48 h of transfection.

### Immunohistochemistry

Bladder cancer and paired normal tissues were obtained from the surgery during January 2007 to November 2011. Tissue samples from 58 patients with age ranged from 44 to 85 years were fixed with 10% formalin and then embedded with paraffin, and tissue microarray for immunohistochemistry (IHC) assay was prepared. Related information of these bladder cancer patients and written informed consents were collected. The study was approved by Ethics committee of Peking University People’s Hospital. For IHC, tissue microarray was dewaxed and hydrated, and were blocked with 3% H_2_O_2_. After washing, the slides were incubated with primary antibodies overnight at 4 °C and then incubated with secondary antibody in the dark for 2 h at room temperature. Antibodies used here was showed in Supplementary Table S1. Diaminobenzene and hematoxylin was added for coloring. The slides were exanimated with ×200 and ×400 objective microscopic and observed by two independent pathologists. Appropriate positive and negative controls were tested in parallel. DEPDC1B overexpression were determined according to tissues staining intensity and staining extent.

### Lentivirus plasmid construction

Primer amplification sequence and shRNA sequence of DEPDC1B gene (5′-gcTGCTAGATTGGTAACGTTT-3′) was designed and stable lentivirus plasmids were obtained from Shanghai Bioscienceres. Co., Ltd (Shanghai, China). Three KD sequences of downstream gene SHC1 were designed as well (5′-TGCCAAAGACCCTGTGAATCA-3′, 5′-CTGAAATTTGCTGGAATGCCA-3′, and 5′-GTAGACATGAGGCTTCGGGAA-3′).

KD expression T24 and EJ cells, overexpression T24 cells and related controls cells were transfected with related lentiviruses (1 × 10^7^ TU/mL × 400 µL) with ENI.S and Polybrene additives in 6 cm dishes. After cultured for 72 h, cell infection rates were observed under microscopic fluorescence. The KD efficiency of shDEPDC1B and shSHC1 were analyzed via quantitative PCR (qPCR) analysis. The assays were performed by triplicate.

### Western blotting

Transfected T24 and EJ cells were lysed with ice-cold Radio-Immunoprecipitation Assay (RIPA) lysis buffer and total proteins were extracted. The concentration of proteins was determined by BCA protein reagent kit (HyClone-Pierce, Logan, UT, USA). western blotting (WB) assays were performed with 20 μg purified proteins. Proteins were separated by 10% SDS-polyacrylamide gel electrophoresis gel and then all were transferred to a polyvinylidene difluoride membrane. Primary and secondary antibodies incubated were described in Supplementary Table S1. Blots were developed using ECL-PLUS/Kit (Amersham, Chicago, IL, USA) and we use glyceraldehyde 3-phosphate dehydrogenase (GAPDH) as normalizing protein.

### RNA extraction and qRT-PCR

Transfected cells in 6 cm dishes with 85% cell density were collected and total RNA was isolated using Trizol reagent (Invitrogen, Carlsbad, CA, USA), and were quantified on Nanodrop 2000/2000C Spectrophotometer (Thermo, Waltham, MA, USA). RNA samples (2.0 μg) were used for reverse transcription of cDNA with a Promega M-MLV kit (Heidelberg, Germany) according to the manufacturer’s instructions. Real-time PCR was conducted with SYBR Premix Ex Taq (Qiagen, The Netherlands, Hilden, Germany) and Mx3000P QPCR System. GAPDH served as inner control. 2^−ΔΔCt^ Method was applied for relative quantitative analysis. Primer sequences used showed in Supplementary Table S2.

### Celigo cell counting assay

Seventy-two hours after the transfection, T24 and EJ cells were collected and seeded into 96-well plates with a cell density of 2000 cells per well. Cells were further cultured in RPMI-1640 medium containing 10% FBS at 37 °C with 5% CO_2_ for 24 h. Cell counting was accomplished by Celigo image cytometer (Nexcelom Bioscience, Lawrence, MA, USA) at day 1, 2, 3, 4, and 5, and the cell proliferation curve was drawn.

### Colony formation assay

T24 cells transfected with DEPDC1B overexpression lentivirus, shSHC1 lentivirus, or DEPDC1B + shSHC1 lentivirus were cultured for 5 days and were seeded in six-well plates, respectively. After growing for 8 days, the colonies were fixed with 4% paraformaldehyde (Sigma, St. Louis, MO, USA) for 30 min and stained with 5 mL GIEMSA solution (Shanghai Dingguo, China) for 15 min. Colonies were graphed with a digital camera and were counted.

### Wound-healing assay

LV-shDEPDC1B- and LV-shCtrl-transfected T24 and EJ cells were seeded a 96-well dish with 5 × 10^4^ cells per well. After growing for 72 h, line wounds across cell layer of each well were made by a 96 wounding replicator (VP scientific, San Diego, CA, USA). Photographs were taken by a fluorescence micrograph at 4 and 8 h post wound-making. Cell migration rate of each group was calculated based on the pictures.

### Cell apoptosis and cell cycle

LV-shDEPDC1B- and LV-shCtrl-transfected T24 and EJ cells were seeded in a six-well dish in triplicate and cultured for 5 days in 37 °C with 5% CO_2_. Cells were collected and washed with 4 °C-cold D-Hanks (pH 7.2 ~ 7.4). After centrifugation at 1300 r.p.m., cells were resuspended by 200 mL 1× binding buffer. For cell apoptosis assay, cells were stained by 10 μL Annexin V-APC solution for 15 min at room temperature in the dark. For cell cycle assay, cells were stained with 1 mL cell staining solution (40× PI, 2 mg/mL : 100× RNase, 10 mg/mL : 1× PBS = 25 : 10 : 1000) for 30 min. FACScan and micropublisher 3.3RTV (Olympus, Tokyo, Japan) were used for analyses. The percentage of the cells in G0–G1, S, and G2–M phases was counted and cell apoptotic rates were assessed.

### Transwell assay

Transfected T24 and EJ cells were resuspended with serum free medium and seeded into upper chamber of a 24-well Transwell migration insert (Corning, NY, USA) with 5 × 10^5^ cell/well. The lower chamber was filled with culture medium containing 30% FBS and incubated for 24 h at 37 °C. The non-invading cells in the upper chamber were removed and the cells adhering to the membrane were fixed in 4% paraformaldehyde and stained with 0.1% crystal violet. Microscopic pictures (×100 and ×200) were collected and the transfer rates were calculated via ImageJ software.

### Human apoptosis antibody array

Total proteins were isolated from shCtrl and shDEPDC1B T24 cells and the proteins’ concentration was detected by a BCA Protein Assay Kit (HyClone-Pierce, Logan, UT, USA). Human Apoptosis Antibody Array (Abcam, Cambridge, MA, USA) were used and biotin-conjugated anti-cytokines and horseradish peroxidase-conjugated streptavidin and chemiluminescent detection reagents were added. Spots on the array membrane were detect by cameras for imaging.

### RNA sequencing

Total RNA samples from stable shDEPDC1B-expressing T24 cells and control sequence T24 cells were isolated by Trizol according to the manufacturer’s guidelines. RNA concentration and A260/A280 values were determined by Thermo NanoDrop 2000 (Waltham, MA, USA) and RNA integrity number (RIN) values were determined using an Agilent RNA 6000 Nano Kit by Agilent 2100 Bioanalyzer (Palo Alto, CA, USA). Human gene chip detection was determined via 3’IVT Plus kit (Affymetrix, Santa Clara, CA, USA) according to the manufacturer’s instructions. Raw data’s quality was assessed and filtered with R studio. Limma package was used for cluster analysis and differential expression of genes assessing, the screening criteria for significant difference genes were as follows: |Fold Change| > 2.0 and false discovery rate < 0.05. Ingenuity Pathway Analysis (IPA) was performed to analysis the canonical pathways, diseases and functions, molecular and cellular processes that are significantly associated with differentially expressed genes (DEGs) in the data sets and |Z score| > 2 is considered to be significant.

### Mice xenograft model

All studies on mice performed in our study were approved by Ethics committee of Peking University People’s Hospital. Specific pathogen-free 4-week-old, female BALB/c nude mice were purchased from Shanghai SLAC Laboratory Animal Co., Ltd (Shanghai, China) and were housed in environmentally controlled conditions (22 °C with a 12 h light/dark cycle). All mice were randomly divided into shDEPDC1B and shCtrl group with four mice in each group. For xenograft model formation, about 4 × 10^6^ stably expressing shDEPDC1B or shCtrl T24 cells were subcutaneously injected into the right back of each mouse (0.2 mL cell suspensions). Mice weight and tumor length and width were recorded two times per week for 6 weeks. The volume of tumors was estimated. Then all mice were isoflurane gas anaesthetized by gas anesthesia system from the Perkin Elmer IVIS Spectrum (Waltham, MA, USA) for in vivo bioluminescence imagine. Finally, all mice were sacrificed and the tumor tissues were weighted and photos were collected.

### Ki-67 immunostaining assay

Mice tumor sections were fixed in 4% paraformaldehyde and, subsequently, paraffin-embedded 5 μm sections were prepared. Sections were immersed in citric acid buffer for antigen retrieval at 120 °C. Ki-67 antibody (Supplementary Table S2) was added for incubating at 4 °C overnight and then secondary antibodies were added as well. Slides were stained by diaminobenzene and then counterstained with hematoxylin. Stained slides were pictured with a microscopic.

### Statistical analysis

The data expressed in the cell experiments as mean ± SD. The differences between two groups were determined with Student’s *t*-test or with parametric or *χ*^2^-test with non-parametric. All analyses were performed on SPSS 7.0 (IBM, SPSS, Chicago, IL, USA) and GraphPad Prism6 (GraphPad Software, La Jolla, CA, USA). *P* < 0.05 was considered statistically significant.

## Results

### DEPDC1B was upregulated in bladder cancer

#### DEPDC1B was upregulated in bladder cancer

**Fig. 1:**
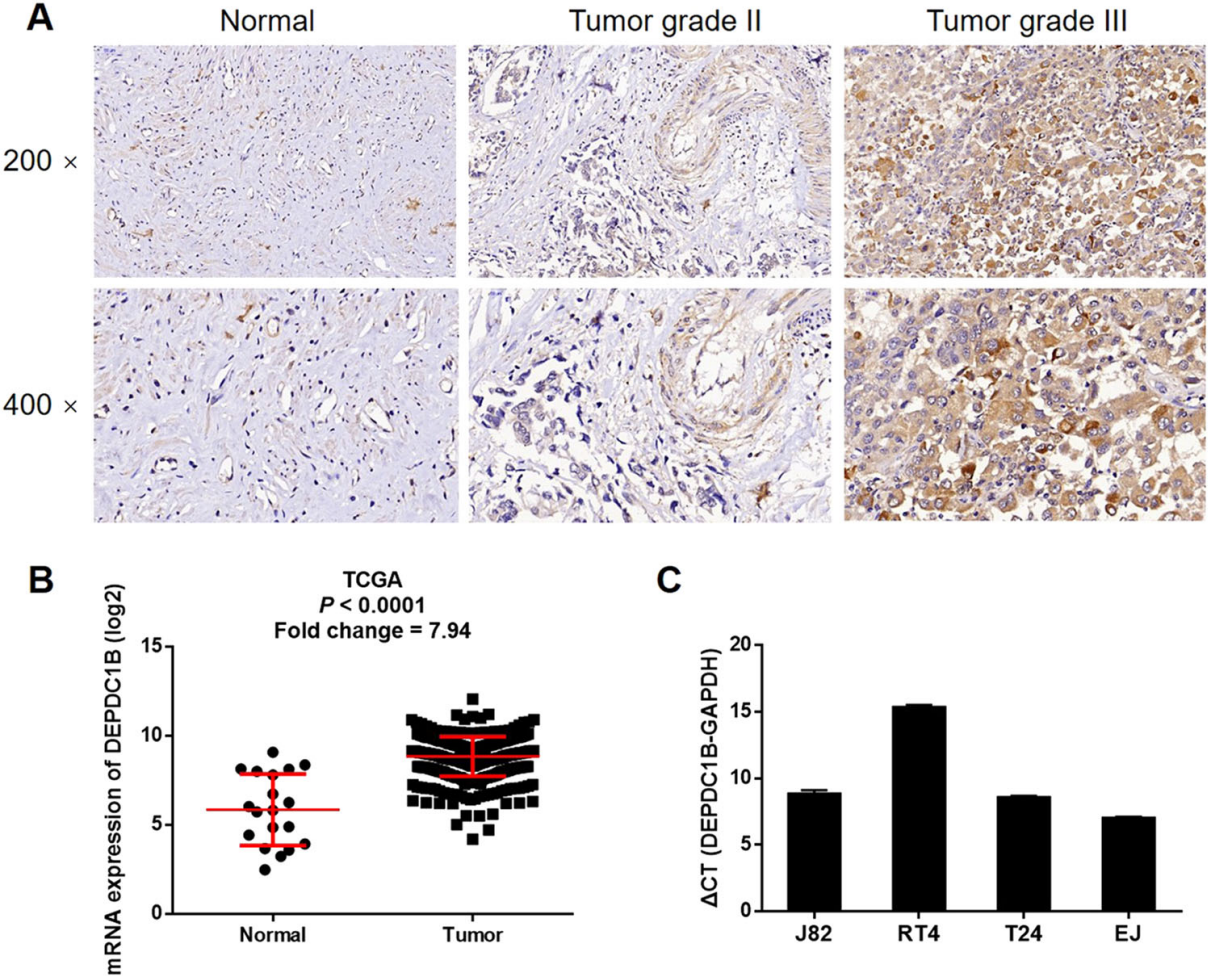
DEPDC1B was upregulated in bladder cancer. **a** The expression of DEPDC1B in tumor tissues of bladder cancer with different malignant grades was detected by IHC and compared with normal tissues. **b** The differential expression of DEPDC1B in bladder cancer tissues and normal tissues was analyzed based on TCGA database. **c** Background expression of DEPDC1B in bladder cancer cell lines including EJ, T24, J82, and RT4 was detected by qPCR. The figures are representative data from at least three independent experiments.

To explore the role of DEPDC1B in bladder cancer, we used IHC to detect the expression levels of DEPDC1B in tumor tissues collected from bladder cancer patients and compared these levels with those from normal tissues. As shown in Fig. 1a and Table 1, the expression levels of DEPDC1B were significantly higher in DEPDC1Btumor tissues than normal tissues (*P* < 0.001) and were also higher in grade III tumor tissues than in grade II tumor tissues. Statistical analysis of the relationship between DEPDC1B expression and the tumor characteristics of patients with bladder cancer also suggested similar results in that high expression levels of DEPDC1B were positively and significantly associated with a more advanced grade of malignancy (*P* = 0.041, Table 2). This association was further confirmed by Spearman rank correlation analysis (Supplementary Table S3), thus indicating the potential role of DEPDC1B in the development and progression of bladder cancer. Gene expression profiling data acquired from The Cancer Genome Atlas also demonstrated the clear upregulation of DEPDC1B expression in bladder cancer tissues (with a fold change of 7.94, *P* < 0.001, Fig. 1b). Moreover, we also used qPCR to determine the background expression of DEPDC1B in various bladder cancer cell lines (EJ, T24, J82, and RT4); this analysis demonstrated high expression levels of DEPDC1B in all cells DEPDC1B (Fig. 1c). Collectively, these results demonstrated that levels of DEPDC1B are elevated in bladder cancer and highlighted the potential role of DEPDC1B as a tumor promotor.

**Table 1:** Expression patterns of DEPDC1B in bladder cancer tissues and normal tissues revealed in immunohistochemistry analysis.

DEPDC1B expression	Tumor tissue		Normal tissue	
	Cases	Percentage	Cases	Percentage
Low	28	48.3%	44	100%
High	30	51.7%	0	-

*P* < 0.001

**Table 2:** Relationship between DEPDC1B expression and tumor characteristics in patients with bladder cancer.

Features	No. of patients	DEPDC1B expression		*P*-value
		Low	High	
All patients	58	28	30	
Age (years)				0.787
≤71	30	15	15	
>71	28	13	15	
Gender				0.002**
Male	49	28	21	
Female	9	0	9	
Tumor size				0.790
<4 cm	25	13	12	
≥4 cm	31	15	16	
Lymphadenopathy				0.352
Yes	6	4	2	
No	37	17	20	
Grade				0.007**
II	19	14	5	
III	39	14	25	
Stage				0.837
1	6	5	1	
2	11	2	9	
3	17	9	8	
4	7	4	3	
T-infiltrate				0.306
T1	10	8	2	
T2	16	5	11	
T3	22	11	11	
T4	3	1	2	

### KD of DEPDC1B inhibited the development and progression of bladder cancer in vitro

**Fig. 2:**
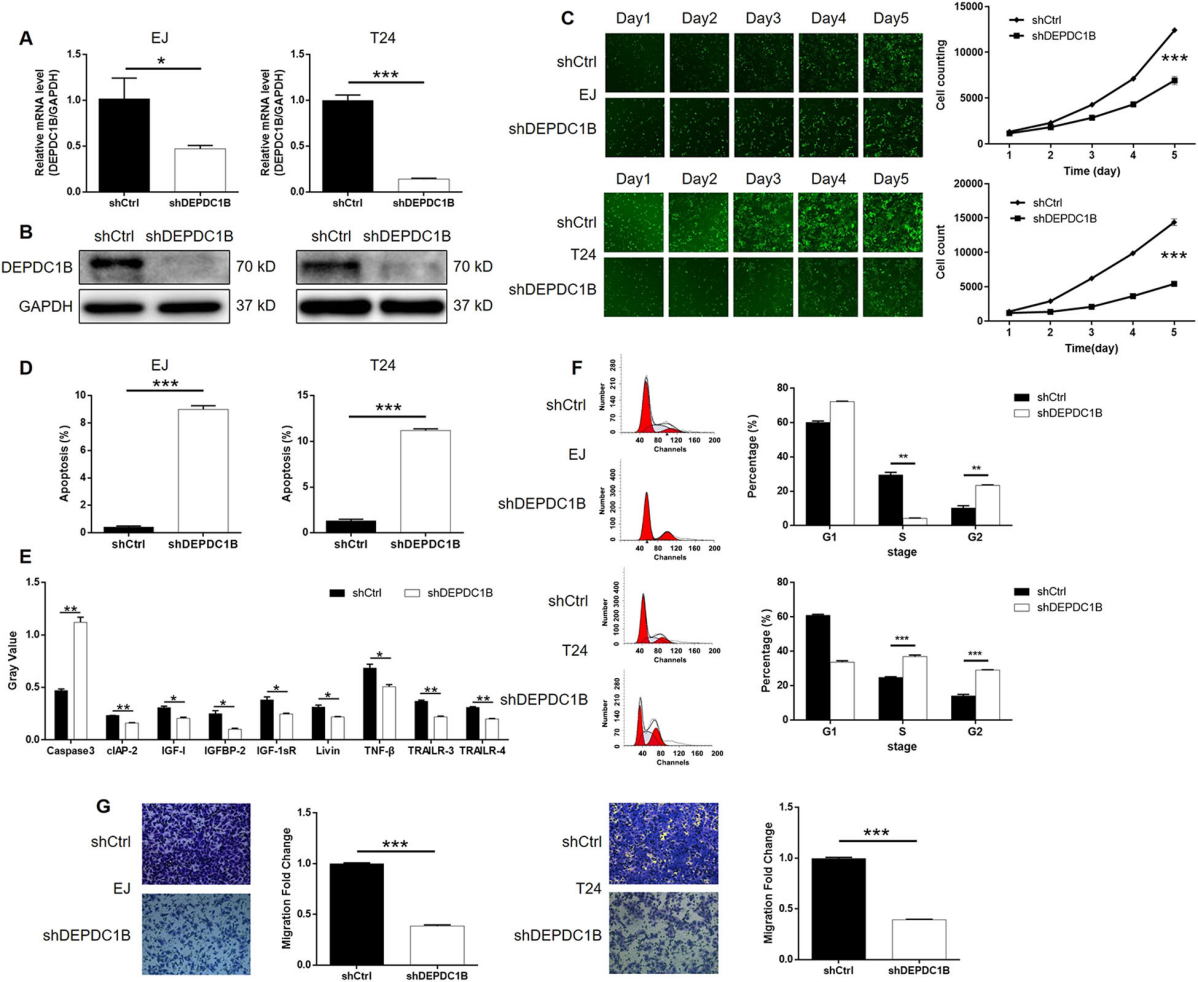
Knockdown of DEPDC1B inhibited development of bladder cancer in vitro. **a**, **b** The efficiency of DEPDC1B knockdown in EJ and T24 cells was assessed by qPCR (**a**) and further verified by western blotting (**b**). **c** The results of Celigo cell counting assay demonstrated that DEPDC1B knockdown significantly inhibited cell proliferation of EJ and T24 cells. **d** The results of flow cytometry showed the significantly promoted cell apoptosis of EJ and T24 cells by DEPDC1B knockdown. **e** Human Apoptosis Antibody Array was used to identify differentially expressed apoptosis-related proteins between T24 cells in shDEPDC1B and shCtrl groups. **f** Flow cytometry was performed to evaluate the effects of DEPDC1B knockdown on cell cycle of EJ and T24 cells. **g** Transwell assay was employed to estimate the effects of DEPDC1B knockdown on cell motility of EJ and T24 cells. The figures are representative data from at least three independent experiments. The data were expressed as mean ± SD (*n* ≥ 3), *P < 0.05, **P < 0.01, ***P < 0.001.

Given the high expression levels of DEPDC1B in bladder cancer cells, we selected EJ and T24 cell lines for the construction of DEPDC1B KD cell models. The transfection efficiencies of lentiviral constructs expressing shDEPDC1B for the DEPDC1B KD of DEPDC1B in EJ and T24 cells, or shCtrl as a negative control, were evaluated by detecting the fluorescence of GFP arising from the lentiviral vector; the transfection efficiency was >80% for both cell lines (Supplementary Fig. S1). Subsequently, we used qPCR to evaluate the KD efficacy of DEPDC1B; data revealed that the expression of DEPDC1B had been reduced by at least 50% DEPDC1B (*P* < 0.001, Fig. 2a). These data were also verified by WB (Fig. 2b). Next, we used Celigo cell counting assays to evaluate cell proliferation; these assays showed that the KD of DEPDC1B significantly inhibited cell proliferation when compared with the shCtrl group (*P* < 0.001, Fig. 2c). Flow cytometry also demonstrated that the KD of DEPDC1B led to an elevation in the proportion of apoptotic cells in EJ and T24 cells, by 18-fold and 9-fold DEPDC1B, respectively (*P* < 0.001, Fig. 2d). To establish how DEPDC1B exerted influence on cell apoptosis, we carried out a Human Apoptosis Antibody Array to identify differentially expressed apoptosis-related proteins between T24 cells in the shDEPDC1B and shCtrl groups. These assays demonstrated the upregulation of Caspase-3 and the downregulation of clAP-2, IGF-I, IGFBP-2, IGF-1sR, Livin, TNF-β, TRAILR-3, and TRAILR-4 (*P* < 0.05, Supplementary Fig. S2 and Fig. 2e). We also investigate the cell cycle n EJ and T24 cells with or without DEPDC1B KD. Despite some differences between the EJ and T24 cells, we found that the KD of DEPDC1B caused significant cell cycle arrest in the G2 phase in both cell lines (*P* < 0.01, Fig. 2f). Transwell assays also showed that the KD of DEPDC1B suppressed the motility of EJ and T24 cells (Fig. 2g). Collectively, these results proved that DEPDC1B KD could suppress the development and progression of bladder cancer in vitro.

### KD of DEPDC1B inhibited tumor growth of bladder cancer in vivo

**Fig. 3:**
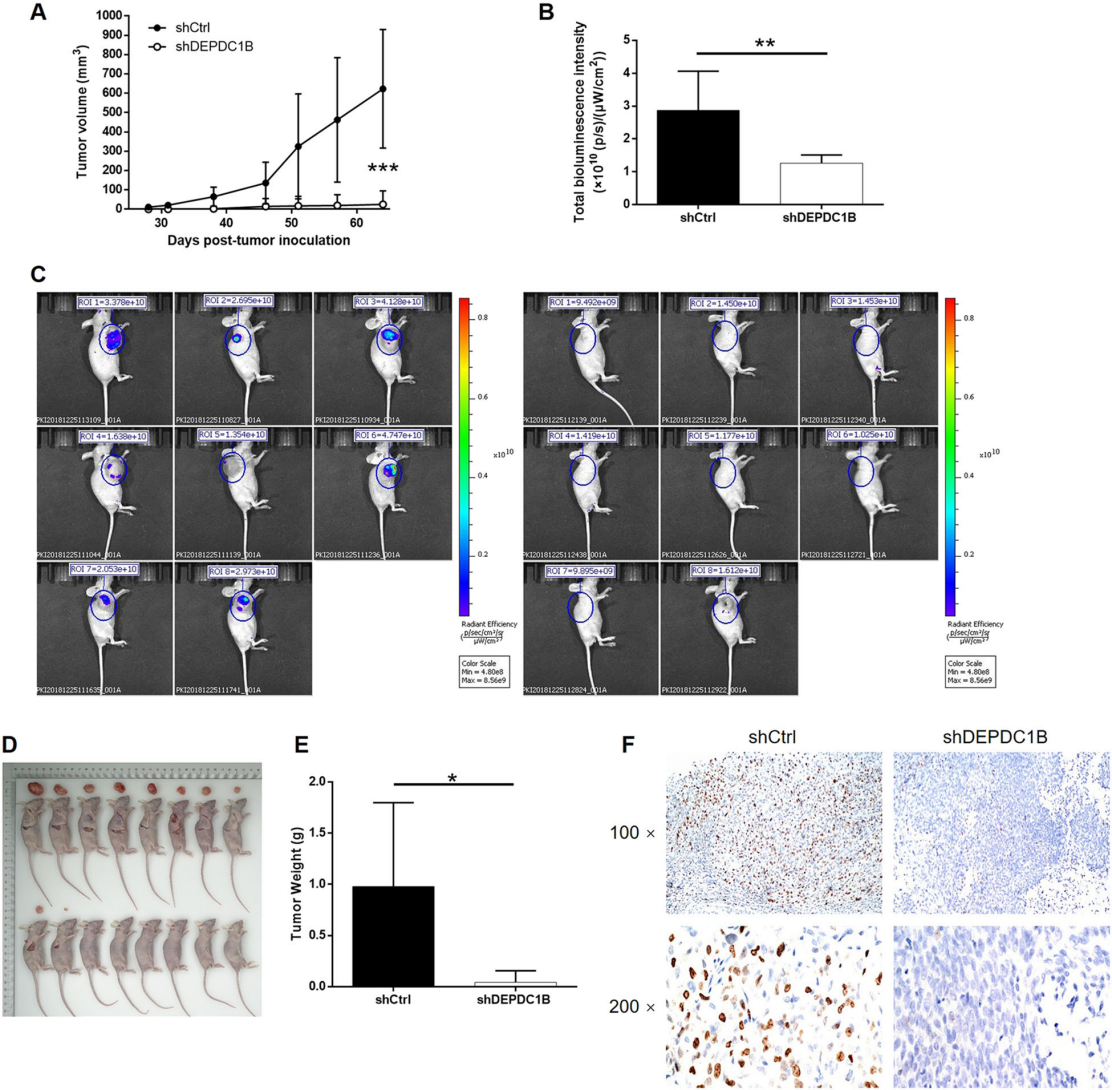
Knockdown of DEPDC1B inhibited tumor growth of bladder cancer in vivo. **a** After the injection of T24 cells with or without DEPDC1B knockdown into mice, the volume of the tumors on mice was measured and calculated at indicated intervals. **b** The bioluminescent imaging of the tumors on mice was performed to assess the size of tumors. **c** The intensity of bioluminescence was calculated and used as the representation of tumor size. **d** Photos of the removed tumors were collected by digital camera. **e** Following the sacrifice of mice, the tumors were removed and weighed. **f** The sections of the removed tumors were subjected to IHC analysis, to detect the expression level of Ki-67. The figures are representative data from at least three independent experiments. The data were expressed as mean ± SD (*n* ≥ 3), *P < 0.05, **P < 0.01, ***P < 0.001.

To further verify the inhibitory effects of DEPDC1B KD on bladder cancer in vivo, T24 cells were transfected with either shDEPDC1B or shCtrl and used to construct a mouse xenograft model by subcutaneous injection. Tumor size was measured in the animals over time and tumor volume was calculated accordingly; these data indicated that tumor growth was significantly suppressed by DEPDC1B KD (*P* < 0.001, Fig. 3a). Bioluminescent imaging of the mouse models, facilitated by the injection of D-luciferin, further showed that smaller tumors were formed by T24 cells in the shDEPDC1B group than in the shCtrl group (*P* < 0.01, Fig. 3b, c). These findings were further verified by tumor weight and digital images, and by examination after the animals were killed (*P* < 0.001, Fig. 3d, e). We also found that tumors in the shDEPDC1B group had a significantly lower Ki-67 index (Fig. 3f); this finding was also consistent with the results described above. Therefore, these studies demonstrated that the KD of DEPDC1B can slow down the growth of tumors in bladder cancer in vivo.

### KD of DEPDC1B may inhibit bladder cancer by regulating SHC1

**Fig. 4:**
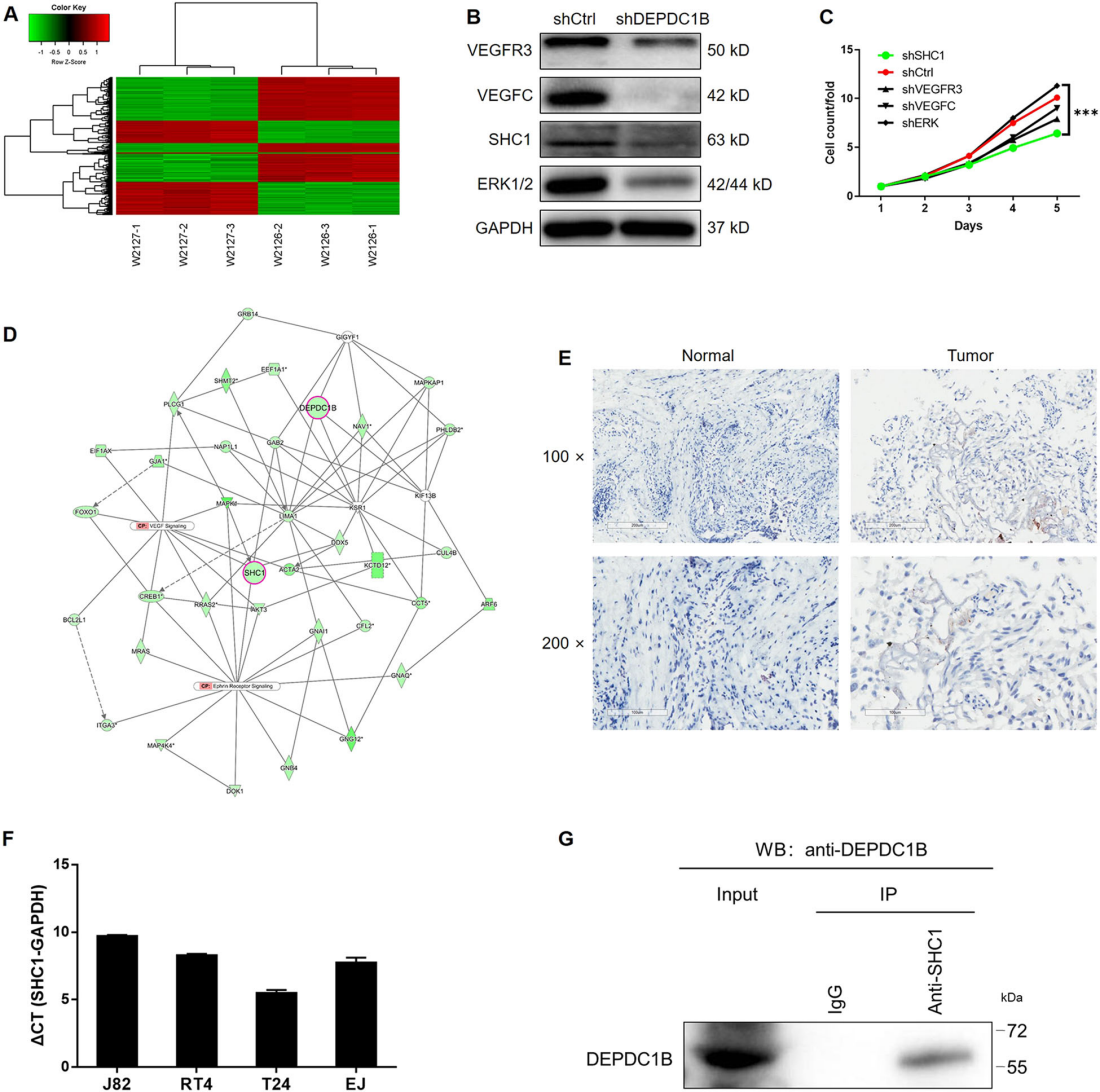
Identification of SHC1 as the potential target of DEPDC1B. **a** Heatmap of the RNA sequencing performed on T24 cells with or without DEPDC1B knockdown, the threshold to identify differentially expressed genes was |Fold Change| ≥ 2.0 and FDR < 0.05. **b** The expression of several selected DEGs in EJ cells with or without DEPDC1B knockdown by western blotting. **c** The effects of several candidates on bladder cancer cell proliferation were evaluated by Celigo cell counting assay. **d** The DEPDC1B-related interaction network was established by IPA analysis and suggested the potential connection between DEPDC1B and SHC1. **e** The expression of SHC1 in tumor tissues of bladder cancer and normal tissues was detected and compared by IHC analysis. **f** The endogenous expression of SHC1 in bladder cancer cell lines was detected by qPCR. **g** The interaction between DEPDC1B and SHC1 was verified by co-IP assay. The figures are representative data from at least three independent experiments. The data were expressed as mean ± SD (*n* ≥ 3), *P < 0.05, **P < 0.01, ***P < 0.001.

Despite the results described above, the regulatory mechanisms underlying the effect of DEPDC1B on bladder cancer still remained unclear. We therefore carried out RNA sequencing on T24 cells with or without DEPDC1B KD (3 v 3). Sequencing identified 1322 DEGs, including 539 upregulated genes and 783 downregulated genes (Fig. 4a and Supplementary Fig. S3A, B). The IPA analysis of diseases and functions indicated that cancer was the most enriched disease by the DEGs (Supplementary Fig. S3C). IPA analysis of canonical signaling pathways revealed the enrichment of several cancer-related signaling pathways, including the molecular mechanisms of cancer and the JAK/Stat signaling pathway (Supplementary Fig. S3D). Bioinformatics analysis identified several DEGs with the highest fold change; these DEGs were then subjected to qPCR and WB for verification in EJ cells. As shown in Figs. S3E and 4B, the mRNA and protein levels of SHC1 were significantly downregulated by the KD of DEPDC1B (*P* < 0.01). Moreover, the effects induced by KD of candidates including SHC1, VEGFC, VEGFR3, and ERK1/2 were evaluated by cell proliferation assay, displaying SHC1 as the strongest inhibitor of bladder cancer proliferation (Fig. 4c). The prediction of a DEPDC1B-related interaction network also revealed the potential connection between DEPDC1B and SHC1 (Fig. 4d). The upregulation of SHC1 in bladder cancer tissues (Fig. 4e) and its highly abundant expression in bladder cancer cell lines (Fig. 4f) were also demonstrated by IHC and qPCR, respectively. Furthermore, a co-immunoprecipitation assay clearly showed the existence of SHC1 in the protein complex precipitated by DEPDC1B antibody, indicating the interaction between them (Fig. 4g). In summary, RNA sequencing indicated that DEPDC1B may regulate the development of bladder cancer by targeting SHC1.

### KD of SHC1 inhibited the development of bladder cancer in vitro

**Fig. 5:**
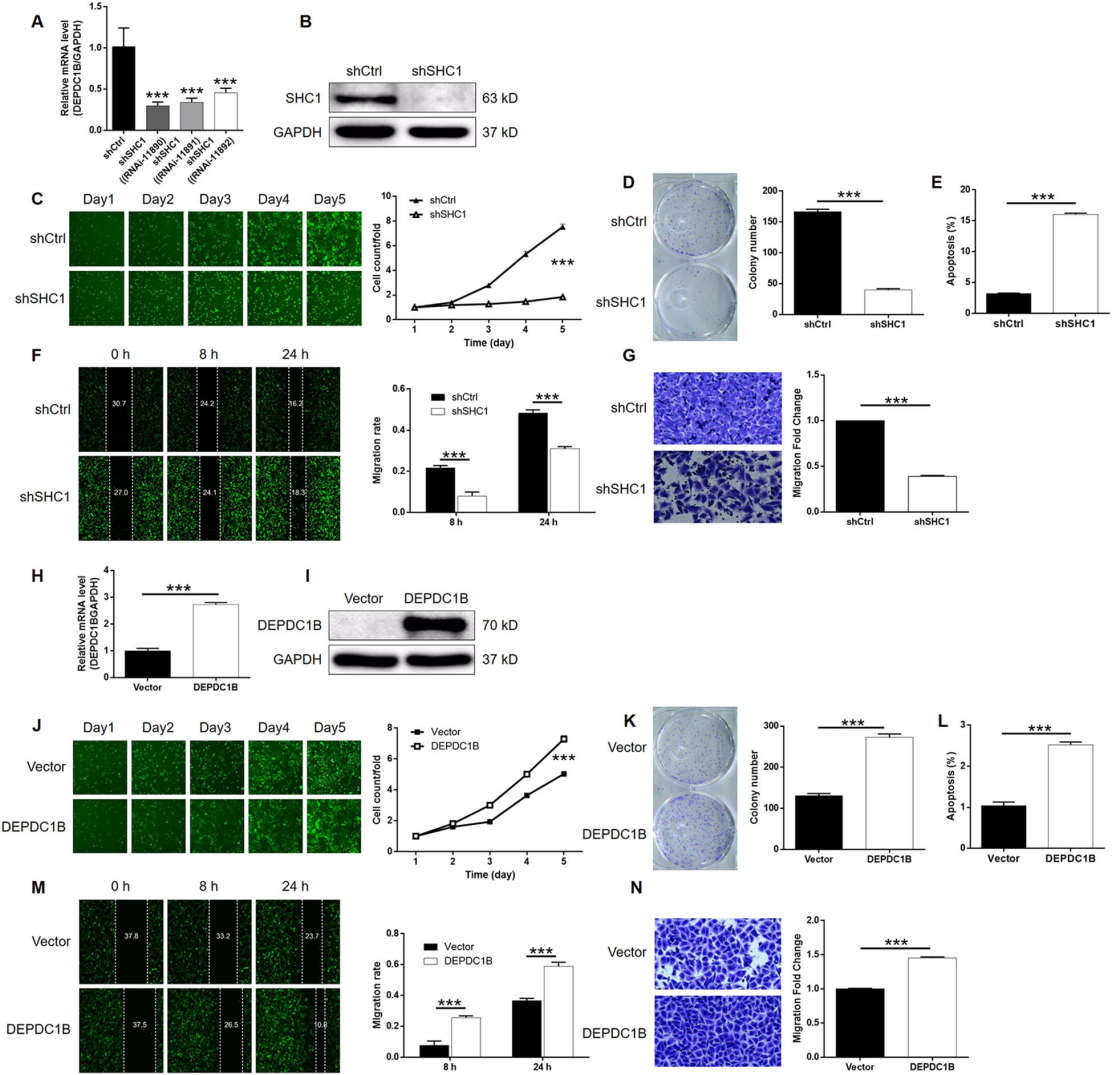
Knockdown of SHC1 inhibited development of bladder cancer in vitro. **a** The knockdown efficiencies of three shRNAs designed for silencing SHC1 were estimated by qPCR and identified RNAi-11890 as the best one. **b** The knockdown efficiency of SHC1 was confirmed by western blotting. **c** Celigo cell counting assay was performed to detect cell proliferation of T24 cells with or without SHC1 knockdown. **d** The effects of SHC1 knockdown on the ability of T24 cells to form colonies were evaluated. **e** The effects of SHC1 knockdown on cell apoptosis of T24 cells were examined by flow cytometry. **f**, **g** Cell migration of T24 cells with or without SHC1 knockdown was assessed simultaneously by wound-healing (**f**) and Transwell (**g**) assays. **h** The overexpression efficiency of DEPDC1B was estimated by qPCR. **i** The overexpression efficiency of DEPDC1B was confirmed by western blotting. **j** Celigo cell counting assay was performed to detect cell proliferation of T24 cells with or without DEPDC1B overexpression. **k** The effects of DEPDC1B overexpression on the ability of T24 cells to form colonies were evaluated. **l** The effects of DEPDC1B overexpression on cell apoptosis of T24 cells were examined by flow cytometry. **m**, **n** Cell migration of T24 cells with or without DEPDC1B overexpression was assessed simultaneously by wound-healing (**m**) and Transwell (**n**) assays. The figures are representative data from at least three independent experiments. The data were expressed as mean ± SD (*n* ≥ 3), *P < 0.05, **P < 0.01, ***P < 0.001.

To further investigate the role of SHC1 in bladder cancer, we constructed a SHC1 KD cell model and verified thus using the method described for DEPDC1B KD DEPDC1B (Supplementary Fig. S4A). Of the three shRNAs designed for SHC1 KD, RNAi-11890 was shown to possess the highest KD efficiency and was therefore utilized in subsequent experiments (*P* < 0.001, Fig. 5a). After further verifying the KD of SHC1 by WB (Fig. 5b), T24 cells with or without SHC1 KD were subjected to Celigo cell counting assays; these assays showed that the knockdown of SHC1 significantly restrained cell proliferation SHC1 (*P* < 0.001, Fig. 5c). We also found that the KD of SHC1 significantly suppressed the colony formation ability of T24 cells (*P* < 0.001, Fig. 5b). As with the DEPDC1B KD, the KD of SHC1 led to a fourfold elevation SHC1 in apoptosis in T24 cells (*P* < 0.001, Fig. 5e). Furthermore, wound-healing and Transwell assays showed that the KD of SHC1 could significantly inhibit the migration ability of T24 cells (*P* < 0.001, Fig. 5f, g). Therefore, the KD of SHC1 exhibited similar inhibitory effects on bladder cancer as DEPDC1B KD.

### KD of SHC1 alleviated the promotion of bladder cancer by DEPDC1B overexpression

**Fig. 6:**
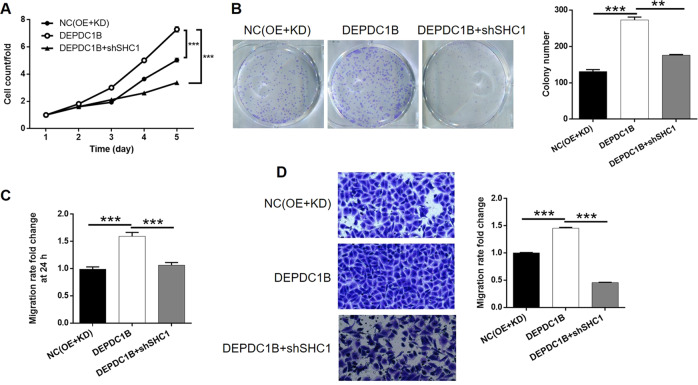
Knockdown of SHC1 alleviated the effects of DEPDC1B overexpression on bladder cancer. **a** Celigo cell counting assay was performed to detect cell proliferation of T24 cells in NC(KD + OE), DEPDC1B, and DEPDC1B + shSHC1 groups. **b**–**d** The synergistic effects of DEPDC1B overexpression and SHC1 knockdown on colony formation (**b**) and cell migration detected by wound-healing assay (**c**), and cell migration detected by Transwell assay (**d**) were assessed. The figures are representative data from at least three independent experiments. The data were expressed as mean ± SD (*n* ≥ 3), *P < 0.05, **P < 0.01, ***P < 0.001.

To investigate the synergistic effect of DEPDC1B and SHC1 on bladder cancer, we constructed T24 cells that overexpressed DEPDC1B, and T24 cells that simultaneously overexpressed DEPDC1B but with the KD of SHC1. The effects of DEPDC1B overexpression on the function of DEPDC1B T24 cells were investigated after detecting the transfection efficiency (>80%, Supplementary Fig. S4B) by fluorescent imaging and verifying the KD efficiency by qPCR and WB (*P* < 0.001, Fig. 5h, i). The overexpression of DEPDC1B significantly promoted the proliferation (*P* < 0.001, Fig. 5j) and colony formation ability (*P* < 0.001, Fig. 5k) of T24 cells; these findings were in stark contrast to those arising from DEPDC1B and SHC1 KD. The extent of apoptosis in T24 cells was also promoted by the overexpression of DEPDC1B; without DEPDC1B overexpression, the rate of apoptosis was low (Fig. 5l). We also found that the overexpression of DEPDC1B significantly promoted the cell migration ability of T24 cells in both wound-healing and Transwell assays (*P* < 0.001, Fig. 5m, n). On the other hand, we also demonstrated successful transfection, DEPDC1B overexpression and SHC1 KD in T24 cells in the DEPDC1B + shSHC1 group (*P* < 0.01, Figure S4C and S5); in this group, we observed an inhibition of cell proliferation DEPDC1BSHC1 (*P* < 0.001, Fig. 6a). Subsequent experiments showed that the effects of DEPDC1B overexpression on colony formation (*P* < 0.01, Fig. 6b) and cell migration (*P* < 0.001, Fig. 6c for wound-healing assay and 6D for Transwell assay) could be attenuated or even reversed by SHC1 KD. These results suggested that DEPDC1B may exert regulatory effects on bladder cancer *via* the regulation of SHC1.

## Discussion

An increasing body of evidence clarified that abnormal overexpression of DEPDC1B in diverse types of cancers, including non-small cell lung cancer, soft tissue sarcoma, cervical cancer, malignant melanoma, and hepatocellular cancer^23–27^. More recently, it was demonstrated that the KD of DEPDC1B inhibits the occurrence of malignant melanoma by inhibiting cell proliferation and inducing cell apoptosis^26^. Moreover, DEPDC1B was found to be able to promote migration and invasion of pancreatic cancer through Rac1/PAK1-LIMK1-Cofilin1 signal pathway^28^. However, the understanding of the relationship between DEPDC1B and human cancers and the underlying mechanism was still in shortage. To the best of our knowledge, this study should be the first one reporting the role of DEPDC1B in bladder cancer.

In this study, we aimed to explore the functions and mechanism of DEPDC1B in bladder cancer. The upregulated expression of DEPDC1B was observed in tumor tissues of bladder cancer and DEPDC1B high expression was found to be associated with advanced malignant grade. Moreover, KD of DEPDC1B significantly inhibited cell proliferation of bladder cancer, and promoted cell apoptosis through the regulation of Caspase-3, clAP-2, IGF-I, IGFBP-2, IGF-1sR, Livin, TNF-β, TRAILR-3, and TRAILR-4. The promotion of DEPDC1B KD on cell apoptosis could also be attributed to the arrest of cell cycle in G2 phase by DEPDC1B KD. Besides, we also found that DEPDC1B overexpression exhibited conversed effects against DEPDC1B KD on cell proliferation and cell motility. All these results recognized DEPDC1B as a tumor promotor in the development and metastasis of bladder cancer. Furthermore, the role of DEPDC1B in bladder cancer was also proved by in vivo experiments, which showed significantly restrained tumor growth of bladder cancer upon DEPDC1B KD.

Through RNA sequencing, the potential downstream of DEPDC1B in the regulation of bladder cancer was screened and SHC1 was identified as the most promising candidate. SHC1 is a member of SHC (Src homolog and collagen protein) gene family, encoding p52ShcA, p46ShcA, and p66ShcA. As typical connector proteins, p52ShcA and p46ShcA play important roles in the transmission of receptor tyrosine kinase signals, which activate Ras signaling pathway through GRB2/SOS axis. p66ShcA was shown to participate in various biological processes such as oxidative stress, cell apoptosis, and cell senescence. More importantly, the relationship between overexpression of SHC, especially SHC1, and tumorigenesis has attracted more and more attention in the recent years^29–31^. Huang et al.^32^ found that high expression of SHC1 was significantly associated with relatively poorer clinical outcomes of hepatocellular carcinoma (HCC) patients. KD of p66Shc could obviously inhibit the development and progression of HCC in vitro and in vivo through modulating Signal Transducer And Activator Of Transcription 3 (STAT3) signaling pathway-mediated regulation of tumor microenvironment^29–31^. Early study has demonstrated that p66ShcA could act as potential prognostic biomarker for more aggressive breast cancer because of its ability to regulate epithelial-to-mesenchymal transition process^33^. A recent work indicated that p52 isoform of SHC1 could function as a key promoter in the 7,12-dimethylbenz(a)anthracene-induced tumorigenesis of breast cancer^34^. In colorectal cancer, SHC1 was identified as a target of tumor suppressor miR-5582-5p to induce cell apoptosis and cell cycle arrest of colorectal cancer cells^35^. Notably, Chao et al.^36^ indicated that the downregulation of SHC1 was the mechanism underlying the regulation of human bladder cancer by RAB14.

Herein, we found that SHC1 KD could significantly inhibit cell proliferation and colony formation of bladder cancer cells, while promoting cell apoptosis. Moreover, SHC1 KD also suppressed cell migration of bladder cancer cells. More importantly, the investigation of the synergistic effects of DEPDC1B and SHC1 on bladder cancer showed that SHC1 KD could alleviate or even reversed the regulation of bladder cancer by DEPDC1B overexpression. All the results showed the role of SHC1 as a tumor promotor in bladder cancer and a potential downstream of DEPDC1B.

In conclusion, we found the upregulated expression of DEPDC1B and SHC1 in tumor tissues of bladder cancer. Both DEPDC1B and SHC1 could act as tumor promotor in the development and progression of bladder cancer, through regulating cell proliferation, colony formation, cell apoptosis and cell migration. More importantly, SHC1 KD could attenuate DEPDC1B overexpression-induced promotion of bladder cancer. Therefore, the regulation of bladder cancer by DEPDC1B through SHC1 make it a potential therapeutic target for bladder cancer treatment.

## Supplementary information

Supplementary figure legends

Table S1

Table S2

Table S3

Figure S1

Figure S2

Figure S3

Figure S4

Figure S5
