# Supplementary material for: DEPDC1B is a tumor promotor in development of bladder cancer through targeting SHC1
Source: Cell Death Dis. 2020 Nov 17;11(11):986. doi: 10.1038/s41419-020-03190-6 (PMC7672062; doi:10.1038/s41419-020-03190-6)
Supplement: Supplementary file 1 — Supplementary figure legends [file 41419_2020_3190_MOESM1_ESM.docx]

**Figure S1.** The observation of fluorescence resulted from the lentivirus-transfected EJ and T24 cells was used to evaluate the efficiency of transfection.

**Figure S2.** Human Apoptosis Antibody Array was employed to identify the differentially expressed proteins between shDEPDC1B and shCtrl groups of T24 cells.

**Figure S3.** (A) Scatter plot of RNA sequencing, the green line represented the differential reference, red dots represented upregulated DEGs, green dots represented downregulated DEGs. (B) Volcano plot of RNA sequencing, the red dots represented the DEGs. (C) The enrichment of the DEGs in IPA disease & function was analyzed by IPA analysis. (D) The enrichment of the DEGs in canonical signaling pathways was analyzed by IPA analysis. (E) The expression of the selected DEGs was detected in EJ cells with or without DEPDC1B knockdown by qPCR. The data were expressed as mean ± SD (n ≥ 3), **P*<0.05, ***P*<0.01, ****P*<0.001.

**Figure S4.** (A) The transfection efficiency of shCtrl and shSHC1 was evaluated by fluorescence imaging. (B) The transfection efficiency of Vector and DEPDC1B construct for DEPDC1B overexpression was evaluated by fluorescence imaging. (C) The lentivirus transfection efficiencies in NC(KD+OE) and DEPDC1B+shSHC1 groups were evaluated by fluorescence imaging.

**Figure S5.** (A) The mRNA expression of DEPDC1B and SHC1 in T24 cells of NC(KD+OE) and DEPDC1B+shSHC1 groups was detected by qPCR. (B) The protein levels of DEPDC1B and SHC1 in T24 cells of NC(KD+OE) and DEPDC1B+shSHC1 groups were detected by western blotting.
