# Supplementary material for: DEPDC1B is a tumor promotor in development of bladder cancer through targeting SHC1
Source: Cell Death Dis. 2020 Nov 17;11(11):986. doi: 10.1038/s41419-020-03190-6 (PMC7672062; doi:10.1038/s41419-020-03190-6)
Supplement: Supplementary file 2 — Table S1 [file 41419_2020_3190_MOESM2_ESM.docx]

Table S1 Antibodies used in western blotting and IHC

| Primary antibodies | Dilution in WB | Source species | Company | Catalog No. |
| --- | --- | --- | --- | --- |
| DEPDC1B | 1:1000 | Rabbit | abcam | ab124182 |
| GAPDH | 1:3000 | Rabbit | Bioworld | AP0063 |
| VEGFR3 | 1:1000 | Rabbit | SAB | 44895 |
| VEGFC | 1:1000 | Rabbit | SAB | 41702 |
| SHC1 | 1:1000 | Rabbit | abcam | ab33770 |
| ERK1/2 | 1:1000 | Rabbit | abcam | ab17942 |
|  |  |  |  |  |
|  |  |  |  |  |
| Primary antibodies | Dilution in IHC | Source species | Company | Catalog No. |
| DEPDC1B | 1:100 | Rabbit | abcam | ab237542 |
| Ki-67 | 1:400 | Rabbit | abcam | ab6721 |
|  |  |  |  |  |
|  |  |  |  |  |
|  |  |  |  |  |
| Secondary antibody | Dilution |  | Company | Catalog No. |
| HRP Goat Anti-Rabbit IgG (WB) | 1:3000 |  | Beyotime | A0208 |
| HRP Goat Anti-Rabbit IgG (IHC) | 1:200 |  | Abcam | Ab111909 |
