# Supplementary material for: DEPDC1B is a tumor promotor in development of bladder cancer through targeting SHC1
Source: Cell Death Dis. 2020 Nov 17;11(11):986. doi: 10.1038/s41419-020-03190-6 (PMC7672062; doi:10.1038/s41419-020-03190-6)
Supplement: Supplementary file 3 — Table S2 [file 41419_2020_3190_MOESM3_ESM.docx]

Table S2 Primers used in qPCR

| Gene | Forward primer sequence (5’-3’) | Reverse primer sequence (5’-3’) |
| --- | --- | --- |
| GAPDH | TGACTTCAACAGCGACACCCA | CACCCTGTTGCTGTAGCCAAA |
| DEPDC1B | CTGAAGTGACCCGCAAACAAA | CTGGTGGGAGATCATTCCATTC |
| MAPK1 | GACTGGACGTGCTCAGACAT | CCTCCAAACGGCTCAAAGGA |
| PLCG1 | GCCTATGCAGATGAACCAGG | CATTCTTTGGCAGATGTCGG |
| SHC1 | TGGTAGACATGAGGCTTCGG | CTGTTTGCGGACTTCTGGAT |
| BCL2L1 | GGAACTCTATGGGAACAATGCA | TGAAGAGTGAGCCCAGCAGA |
| RRAS2 | GAGGCATCAGCAAAGATTAGGA | GGTTCTGGTGAAGGAGGACATT |
| FOXO1 | ACCCAGCCCAAACTACCAA | ACTGACTCATACCTCCATAACTCG |
| ACTA2 | GCTGTTTTCCCATCCATTGTG | CTTTTGCTCTGTGCTTCGTCA |
| IRS1 | GGTGGATGACTCTGTGGTGG | GGACGCTGATGGGGTTAGAG |
| MRAS | ACAACCTCCCCACATACAAGC | GGGTCATAGTCAGGCACAAAGAT |
| AKT3 | TCCTTCCAGACAAAAGACCG | GAATGTAGATAGTCCAAGGCAGAG |
| FRS2 | ACTCCAGGATTTGCTGCTCA | GGATGTCTGCTTGACGGATG |
| ATM | AGATAGTTTGTATGGCTGTGGTGG | GCGTGTTGATGAGGGGATTG |
| VEGFA | CTTGCCTTGCTGCTCTACCT | TTCGTGATGATTCTGCCCTC |
| VEGFC | CTGCCAGCAACACTACCACA | CCAGCATCCGAGGAAAACATAA |
|  |  |  |
|  |  |  |
|  |  |  |
|  |  |  |
|  |  |  |
