# Supplementary material for: DEPDC1B is a tumor promotor in development of bladder cancer through targeting SHC1
Source: Cell Death Dis. 2020 Nov 17;11(11):986. doi: 10.1038/s41419-020-03190-6 (PMC7672062; doi:10.1038/s41419-020-03190-6)
Supplement: Supplementary file 4 — Table S3 [file 41419_2020_3190_MOESM4_ESM.docx]

Table S3 Relationship between DEPDC1B expression and tumor characteristics in patients with bladder cancer analyzed by Spearman rank correlation analysis

| Tumor characteristics | index |  |
| --- | --- | --- |
| Grade | Pearson correlation | 0.355 |
|  | Significance (two tailed) | 0.006** |
|  | n | 58 |
| Gender | Pearson correlation | 0.414 |
|  | Significance (two tailed) | 0.001** |
|  | n | 58 |
