# Supplementary figures and images for: DEPDC1B is a tumor promotor in development of bladder cancer through targeting SHC1
Source: Cell Death Dis. 2020 Nov 17;11(11):986. doi: 10.1038/s41419-020-03190-6 (PMC7672062; doi:10.1038/s41419-020-03190-6)

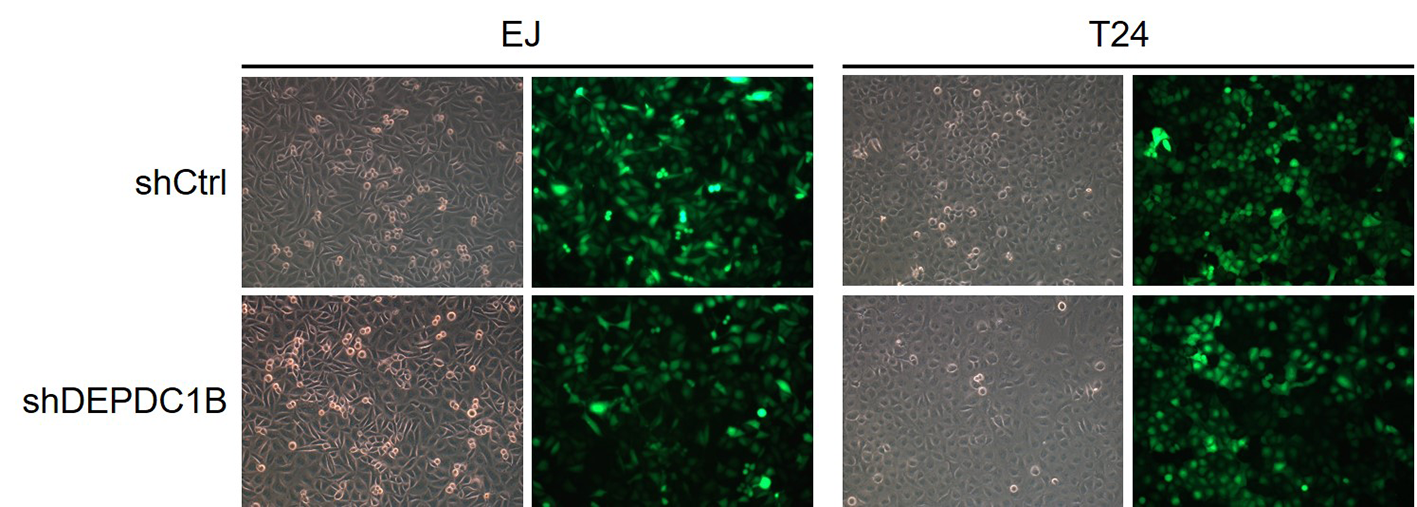

Supplement: Supplementary file 5 — Figure S1 [file 41419_2020_3190_MOESM5_ESM.tif]

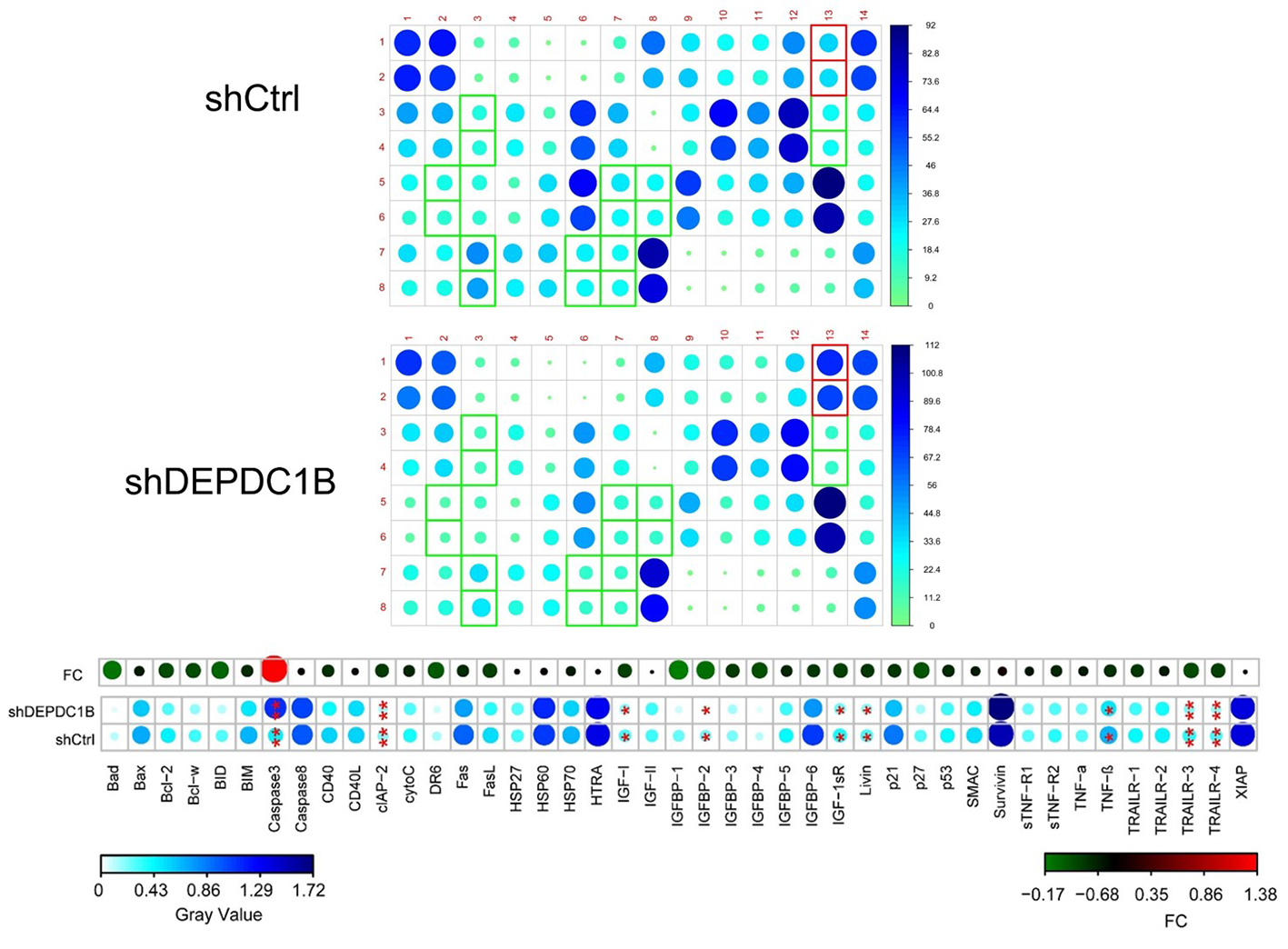

Supplement: Supplementary file 6 — Figure S2 [file 41419_2020_3190_MOESM6_ESM.tif]

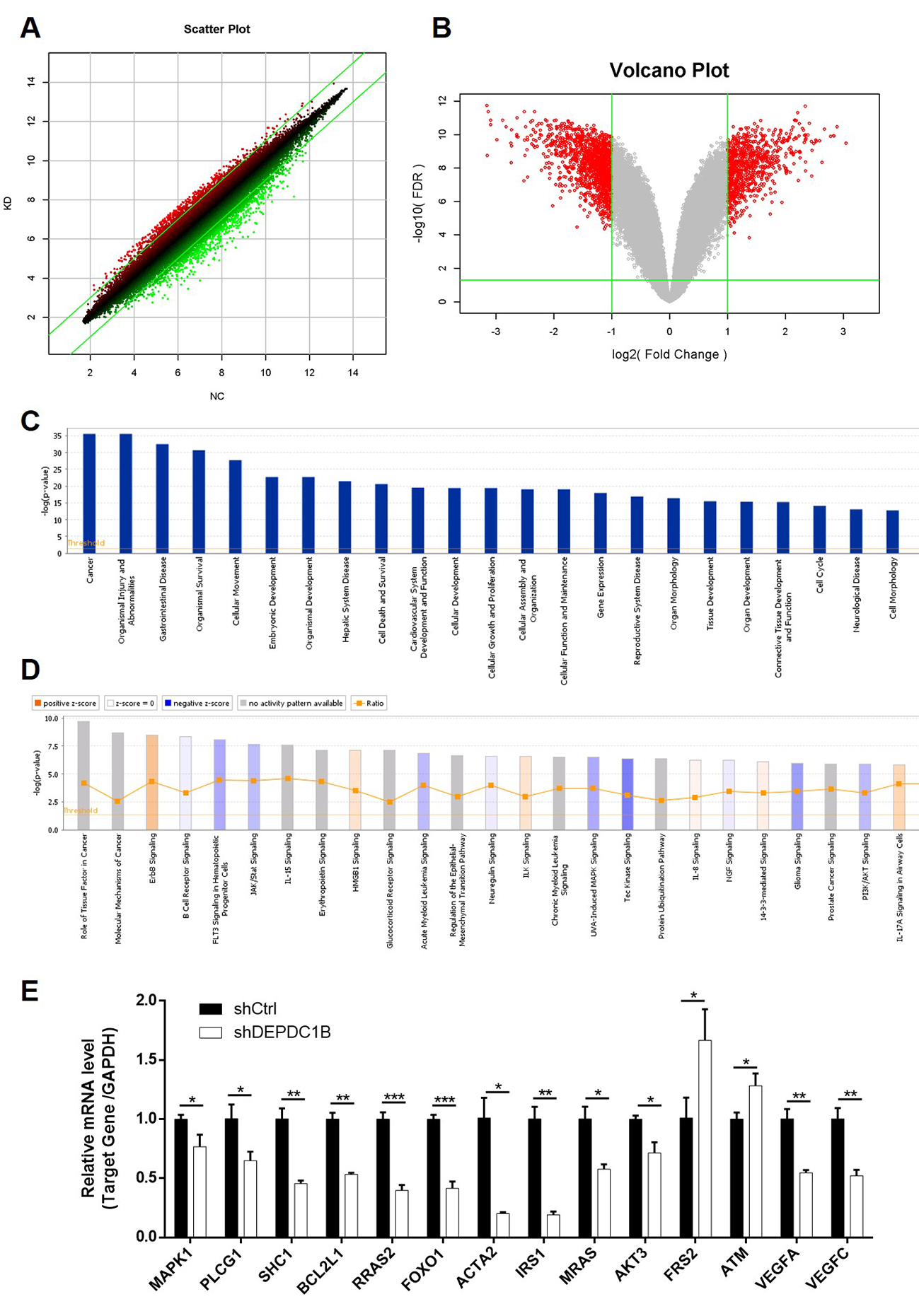

Supplement: Supplementary file 7 — Figure S3 [file 41419_2020_3190_MOESM7_ESM.tif]

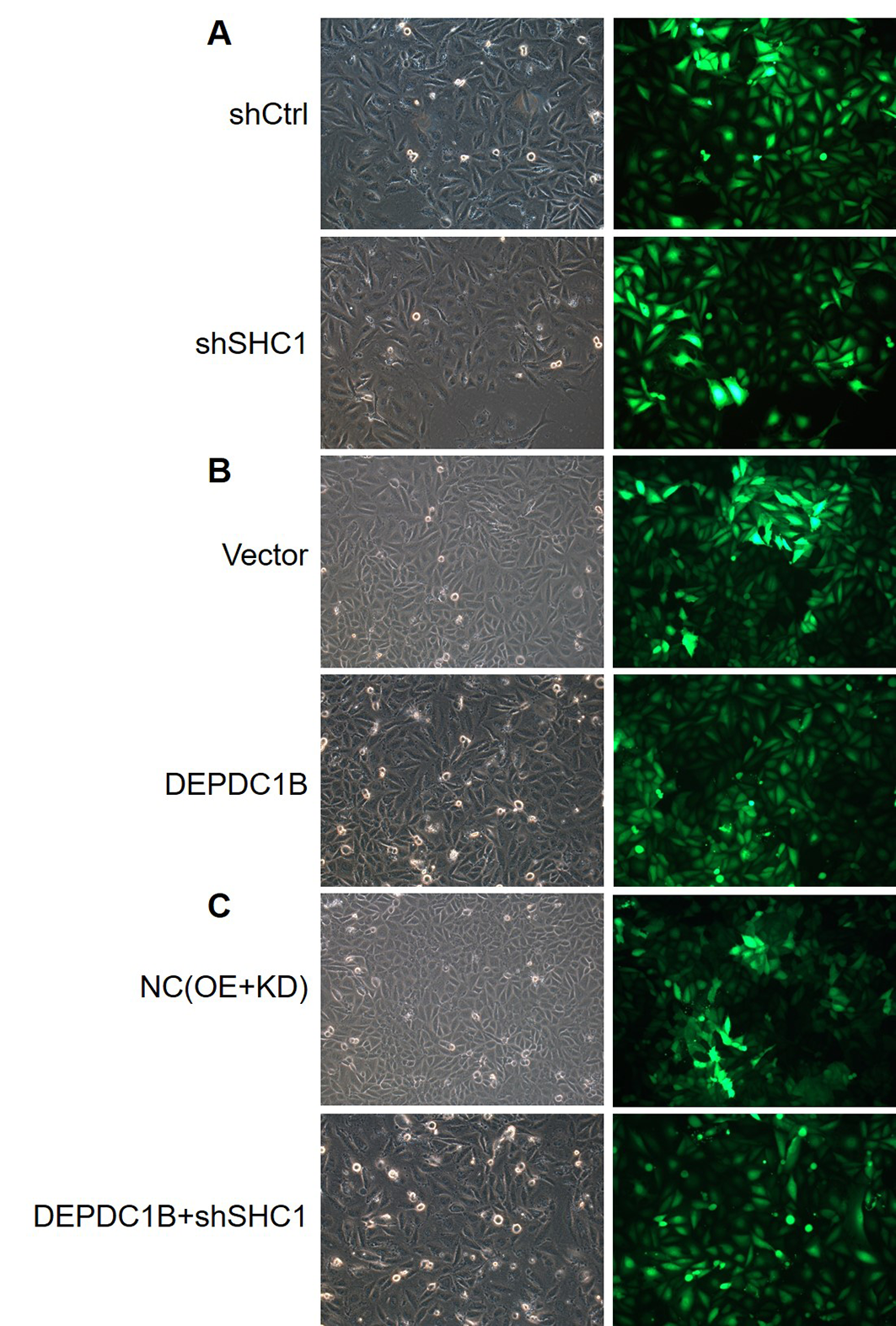

Supplement: Supplementary file 8 — Figure S4 [file 41419_2020_3190_MOESM8_ESM.tif]

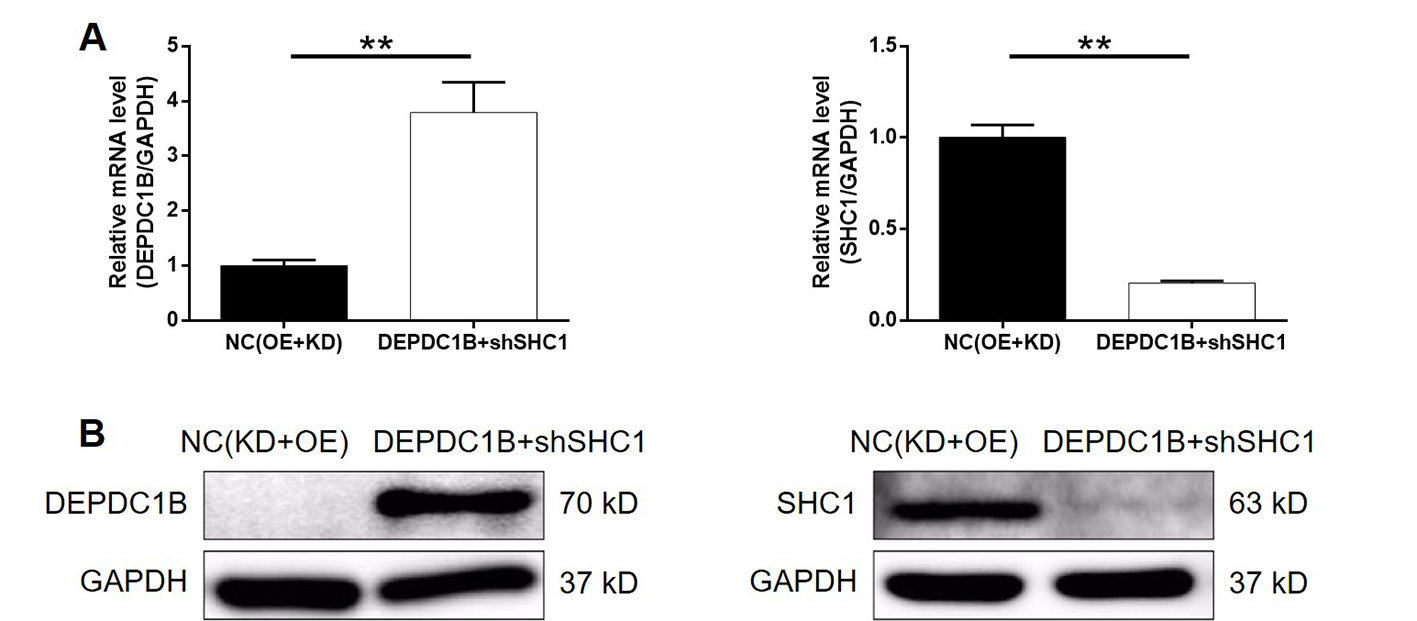

Supplement: Supplementary file 9 — Figure S5 [file 41419_2020_3190_MOESM9_ESM.tif]
